# Relationships between Social Networking Sites Use and Self-Esteem: The Moderating Role of Gender

**DOI:** 10.3390/ijerph191811462

**Published:** 2022-09-12

**Authors:** Cecilia M. S. Ma

**Affiliations:** National Institute of Education, Nanyang Technological University, 1 Nanyang Walk, Singapore 637616, Singapore; cecilia.ma@nie.edu.sg

**Keywords:** social networking sites, gender, self-esteem, adolescents, online activities

## Abstract

With the prevalence of the internet, there is growing attention on the impacts of social networking sites use among adolescents. The purpose of this study was to explore the relationships between different types of online activities (i.e., information searching, social interaction and entertainment) and self-esteem. It examined whether the relationships vary across gender. One hundred and ninety-three students (57.5% males; *M*_age_ = 13.33, *SD*_age_ = 1.58) participated in the present study. Unexpectedly, the associations between online activities and self-esteem were not significant (*p* > 0.05). Path analysis showed gender moderated the relationships between social interaction activities and self-esteem. Females reported higher levels of engagement in social interaction activities and self-esteem than their male counterparts. The present study shows the importance of assessing different types of online activities as a predictor for understanding the impact of social media use among adolescents.

## 1. Introduction

With the prevalence of the internet and smartphone, social networking sites (SNSs) have been increasingly popular over the past decades. As of July 2022, over five billion people use the internet; while nearly 4.7 billion are active social media users [1]. Compared to other countries, Asian people were most frequent social media users (over 2 billion) [2]. In this context, *Facebook* is the most frequent use SNS (almost 3 billion), followed by *YouTube* (almost 2.5 billion) and *Instagram* (almost 1.5 billion) [3].

Given the increased popularity of the SNSs, scholars began to explore the potential impacts of SNS use. Excessive SNS use is defined as “being overly concerned about social media, driven by an uncontrollable motivation to log on to or use social media, and devoting so much time and effort to social media that it impairs other important life areas” ([4], p. 4054). A review study suggested that SNS use may pose threats to adolescent mental health, such as depression, anxiety, poor academic performance, sleep deprivation and other behavioral problems, [5]. In particular, one prominent line of research explores the impact of SNS use on self-esteem.

Self-esteem is described as an individual’s overall evaluation of his or her own worth [6]. A growing body of research suggested that self-esteem is an important predictor of well-being [7,8] and life satisfaction [9]. The impact of self-esteem on well-being is further supported in meta-analyses [10,11].

As a part of self-concept [12], researchers began to explore the role of self-esteem on SNS use [13,14]. First, the linkage between SNS use and self-esteem is associated through the cognitive process [15]. Individual’s self-esteem might be enhanced when they received “*likes*” on their posted content [16] or developed a sense of connectedness through interpersonal communication [17,18,19]. Second, adolescents with emotional and adjustment problems are likely to engage in SNS as a self-soothing experience to escape from reality [20,21]. Individuals, especially those with low self-esteem, can benefit from this medium which allows them to socialize comfortably and seek social recognition [22,23]. The evidence of developing social capital among low self-esteem individual has been support by a recent meta-analysis [24]. This has been further demonstrated by an experimental study [25] which showed that SNS use (i.e., *Facebook*) is associated with a short-term increase in self-esteem.

While SNS use becomes an essential part of our life, scholars start to test the impact of SNS use. Past studies have shown that SNS use is a significant predictor of poor well-being [20,26]. For example, the use of SNS (e.g., *Facebook*) is negatively associated with self-esteem among young adults [13,27,28]. Using a four-year cross-lagged longitudinal design, Steinsbekk et al. [29] found that passive SNS use (e.g., browsing others’ profile) is negatively predicted self-esteem among a sample of Norwegian adolescents. Notably, this relationship is more salient among adolescent girls who frequently engage in social comparison on social media.

Although the negative impacts of SNS use on self-esteem have been found, researchers argue that SNS use maybe beneficial to well-being [30]. Studies found that SNS use is associated with better subjective well-being (e.g., life satisfaction) and less depressive symptoms and loneliness [31,32]. For instance, Jelenchick et al. [33] found the benefits of SNS use for patients with depressive symptoms. Similar results are shown in self-esteem. Researchers showed that intense SNS use is associated with higher self-esteem [34,35]. Furthermore, cross-sectional [36,37] and experimental studies [24,38] suggested that SNS use is associated with momentary positive changes in self-esteem. In particular, a weak and negative effect of SNS use on social self-esteem, ranging from −04 to −09 has consistently been found in meta-analytic work [25,39]. Taken together, the aforementioned studies showed the relations between SNS use and well-being remains unclear. Researchers noted that the prevalence of a cross-sectional design may limit our understanding of the temporal relationships among variables in the field of SNS use research [39,40,41].

Perhaps, the mixed results may indicate the limitations of the existing literature. First, the available findings have exclusively focused on a specific social networking site, such as *Facebook* [42,43,44], *Instagram* [45] or general social media use [46]. Researchers noted that the well-being impact of SNS use may vary depending on the motives and content of the SNS use [7,46]. This is supported by Carlson et al. [47] who suggested that people engage in SNS use mainly for three different purposes, such as information searching, entertainment, and social interaction. To date, little is known whether the impact of SNS use varies by different types of online activity. Researchers call for the need to assess the consequences of different types of online activities in order to comprehensively capture the whole array of SNS use in this area of research [29,48].

Moreover, studies have mostly focused on the relationships between SNS use and psychological vulnerabilities. Researchers argued the need to take other potential confounders into account [49,50]. Past studies have controlled socio-demographic factors as covariates, such as age, gender, and socioeconomic status, but little has explored the moderating effect of these variables. Researchers noted that investigating the effects of these variables may extend the existing literature [51,52]. The present study attempts to fill this research gap by testing the moderating role of gender.

Past studies show that gender may moderate the relationships between internet addiction and psychological outcomes [53,54]. Adolescents are likely to use the social media platforms to gain social support and to express their negative emotions freely with other users [55]. Compared to males, females’ self-esteem is more social-oriented [56]. Females are more likely to develop social media addictive symptoms [57] and actively engage in online social interaction activities [22]; while males are more inclined to participate in solitary activities, such as online gaming [58,59]. It is noteworthy that these associations were more prevalent in younger social media users compared to their older counterparts [60]. Individuals with emotional and adjustment problems are sensitive to interpersonal relationships and therefore may prefer to socialize in virtual contexts in which they feel less threatened [26,61,62]. To date, little is known about the moderating role of gender underlying these relations. Hence, the present study investigates whether the relationships between different types of SNS use and self-esteem differ by gender.

To summarize, the present study aimed to extend the literature by exploring the relationships between different types of online activities (i.e., information searching, social interaction and entertainment) and self-esteem. Moreover, it tested whether these relationships vary across gender. The present study attempts to address the following research questions:

RQ 1: Does SNS use relate to self-esteem? If so, how different types of online activity (i.e., information searching, social interaction and entertainment) are related to self-esteem? Based on the literature, it appears that all online activities will have negative effects on self-esteem.

RQ2: Does gender moderate the relationship between SNS use and self-esteem?

Compared to males, the potential effects of online activities on self-esteem will be much stronger among females.

**Figure 1:**
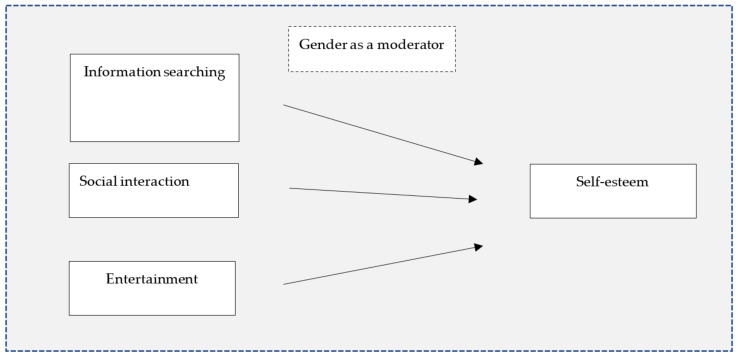
A hypothesized model of the present study.

A conceptual model of the present study is shown in Figure 1.

## 2. Method

### 2.1. Participants

193 students (111 males = 57.7%, 1 student did not report his/her gender information) from two secondary schools in Hong Kong participated (Table 1). These schools were recruited through a service-learning project which aimed at addressing community-identified needs by providing meaningful services activities [63]. The mean age is 13.33 (*SD* = 1.58), ranging from 12 to 19 years old (males: *M*_age_ = 13.53, *SD*_age_ = 1.67; female: *M*_age_=13.05, *SD*_age_ = 1.43). Sixty-one percent of the participants received financial aid either from their schools or the government (*n* = 116, 2 students did not report this information). One hundred and fifty-nine students (83.2%) were born in Hong Kong. One hundred and thirty-one students (69.7%) were from intact families. The study was conducted in Spring–Summer 2019.

### 2.2. Measures

#### 2.2.1. Online Activities

Following the findings of Carlson’s study [47], online activities were assessed by eight items focusing on three areas: *information searching* (two items, “google search”, “chatroom”, Cronbach’s alpha = 0.67), *entertainment* (four items, “watching *YouTube*”, “download software”, “online gaming”, “online shopping”, Cronbach’s alpha = 0.70), *social interaction* (two items, “social media platforms”, such as “*Instagram*, *Facebook*, *Snapchat*” and communication, such as “email, *WhatsApp*, *WeChat*”, Cronbach’s alpha = 0.75). Participants rated on a five-point scale, ranging from 0 (never) to 5 (more than seven times per week), with high scores indicating high levels of engagement in a specific type of online activities.

#### 2.2.2. Self-Esteem

The 10-items Rosenberg self-esteem scale [64] was adopted to measure students’ perceived self-esteem. Participants rated on a four-point scale, ranging from 1 “strongly disagree” to 4 “strongly agree”. A higher overall score indicates a higher level of self-esteem. An example item “I take a positive attitude toward myself”. The present sample showed an acceptable internal consistency (Cronbach’s alpha = 0.78).

### 2.3. Procedures

This study was approved by the university’s Institutional Review Board. Students anonymously completed a paper-and-pencil-based questionnaire with demographic information (e.g., age, gender, place of birth, receiving financial aids) after obtaining the informed consent from the school principals, teachers and parents. In general, it took around 15 min to complete the questionnaires.

### 2.4. Data Analysis

Descriptive statistics and correlations among the main variables were computed via IBM SPSS 28.0 version. Independent *t*-test and chi-square preliminary test were performed to assess the relationship between independent variables and dependent variables. Then, path analysis was conducted to investigate the moderating role of gender on the relationships between online activities and self-esteem using *Mplus* 8.7 version. Past studies [65,66,67,68] showed the effects of age and socio-economic status on SNS use, therefore they were included in the analyses as covariate. Missing data were less than 1%. Prior to path analysis, normality of all observed variables is assessed. Results showed that the data are normally distributed (skewness and kurtosis between −2.0 to 2.0) [69], therefore maximum likelihood (ML) was used to estimate the model (Table 2). To evaluate the model fit, several indices were used, including the Chi-square values (*χ*^2^), the comparative fit index (CFI), the Tucker–Lewis fit index (TLI), the root mean square error of approximation (RMSEA) and the standardized root-mean-square residual (SRMR). Researchers noted that CFI and TLI above 0.90 and RMSEA and SRMR below 0.08 indicated a good model fit [70].

## 3. Results

### 3.1. Descriptive Statistics

Results of chi-square tests of independence and independent *t*-test showed no significant gender differences regarding their demographic and family background, except in age (*p* < 0.05, Table 1). Descriptive statistics and correlation coefficient among the variables were shown in Table 2. First, all online activities were moderately related, ranging from 0.42 to 0.54. Second, overall internet use and different types of online activities were not significantly related to self-esteem (Table 2). Results of independent *t*-test showed no significant gender differences in all variables, except in entertainment online activities (*p* < 0.05, Table 3). This indicates females were more likely to use social media for entertainment purpose than males while both genders shared similar usage patterns in other online activities.

### 3.2. Confirmatory Factor Analysis

Confirmatory factor analysis (CFA) was performed to test the factor structure of the measures. The factor structure of three types of online activities (*χ*^2^ = 34.677, *df* = 16, *p* < 0.01; RMSEA = 0.079 (CI = 0.042–0.115); SRMR = 0.051; TLI = 0.905; CFI = 0.946) and self-esteem (*χ*^2^ = 44.69, *df* = 15, *p* < 0.01; RMSEA = 0.069 (CI = 0.026–0.108); SRMR = 0.046; TLI = 0.941; CFI = 0.968) fit the data well. The reliability of one of the online activities (information searching) was somewhat low (α = 0.67) but can still be considered as acceptable (Cronbach α = 0.70) [71]. In general, the reliability of both scales reached at an adequate level, ranging from 0.67 to 0.78.

### 3.3. The Moderating Role of Gender

To test the moderating role of gender on the relations between online activities on self-esteem, path analysis was conducted. Following the suggestions by Dearing and Hamilton [72], all predictors were standardized to reduce the chance of multicollinearity. Gender (*β* = −0.52, *p* < 0.01) and its interaction effect with social interaction (*β* = 0.53, *p* < 0.01) were significantly related to self-esteem when controlling for age and socioeconomic status (*χ*^2^ = 0.00, *df* = 0, *p* < 0.01; CFI = 1.0; TLI = 1.0; SRMR = 0.00; RMSEA = 0.00). All antecedent variables explained 13% of the variance in self-esteem, suggesting that three different types of online activity and gender explained 13% of the variance in self-esteem (Table 4).

### 3.4. Analyses of Covariance (ANCOVAs)

**Figure 2:**
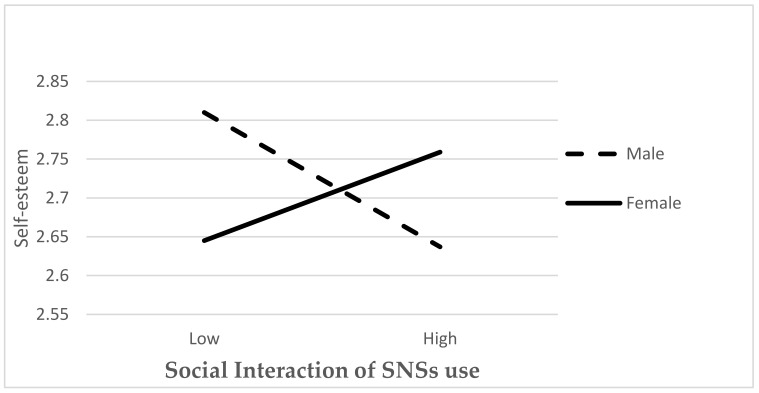
The moderating effect of gender on the relationship between social interaction of SNS use and self-esteem.

Three univariate analyses of covariance (ANCOVAs) were performed to test the differences in online activities among participants of different demographic (i.e., gender) and psychological characteristics (i.e., self-esteem) while controlling for age and socioeconomic status. Results of ANCOVAs revealed a significant gender difference in the relation between social interaction use of SNS and self-esteem, *F*(1,1818) = 4.385, *p* = 0.04, η2 = 0.02). The effect size of this difference was small [73]. While no significant difference was found in other online activities (information searching: *F*(1,1178) = 0.414, *p* > 0.05; entertainment: *F*(1,1180) = 2.284, *p* > 0.05). Results demonstrated that females were likely to engage in social interaction activities and reported high self-esteem; while males reported lower levels of engagement in social interaction activities and self-esteem (Figure 2).

## 4. Discussion

The purpose of the present study was to test the moderating role of gender on the relationship between SNS use and self-esteem. First, a significant interaction effect of gender X SNS on self-esteem was found. Specifically, females were more likely to engage in online social interaction activities and reported higher self-esteem compared to their male counterparts. This was consistent with the past findings consistently showed that females were more addictive to social media use [74,75] and gain online social popularities [76] than males. Empirical evidence shows that social motive was a significant predictor to social media addiction [77]. Perhaps, females may benefit from SNS use with the intention to satisfy their social needs, and thus reported higher self-esteem. This is in line with research showing the positive outcomes of the SNSs use, such as psychological well-being [78,79] and academic experiences [80]. Clearly, future research should explore whether satisfaction of social needs is associated with SNSs use, and how this relation differs by gender and other predisposing factors, e.g., personality, social skills.

Contrary to the past research, the relations between different online activities and self-esteem are not significant. This might be related to students’ characteristics. The present study involved students who were mostly from low school banding (i.e., students with low academic abilities) [81]. In particular, they were from low socio-economic status families (over 60% received financial aids) and studied in the two districts, Tuen Mun and Yuen Long, with the highest poverty rates [82]. Past studies show the influence of socio-economic status on the linkage between the SNS use and well-being [52,83] and self-esteem [41,67]. Perhaps, their family background may be a stronger predictor of self-esteem compared to SNS use. Given the prevalence of SNS use, researchers argued that other potential factors (e.g., online social comparison, personality, nature of SNS use) may be more influential in predicting individual well-being [18,84,85]. Future research should investigate how these factors moderate the relationships between online activities and self-esteem.

Another explanation of these unexpected results may be related to the social network size. Lim et al. [86] found that the negative effects of SNS use on self-esteem was significant only when social network size (less than 150 individual) was small. Young adults and adolescents are frequent SNS users who consider this as part of their daily life. As such, it is not surprise they have a larger social network compare to other population group. In particular, they might not be able to distinguish between “*real*” and “*virtual*” friendship [87]. This has been confirmed by Apaolaza et al. [88] who found that the effects of SNS use on social self-esteem were mediated by the quality of online interpersonal relationships among a sample of Spanish adolescents. Future research may explore the role of social network size regarding the relationship between SNS use and self-esteem.

The present study extends the literature by showing the moderating role of gender on the relationships between online activities and self-esteem. It demonstrates the impact of online activities may depend on “gender”, despite its small effect as shown in the present study. This contributes to the notion on the well-being effects of internet use by considering the participants’ demographic background (e.g., gender). As the internet can provide access to information and social resources, students are likely to use this medium to build social capital, satisfy their social needs and compensate their real-life social relationships by gaining positive feedback and popularity in this virtual context [22,89]. Educators may need to pay more attention to this group who are more susceptible to peer pressure [90] and use social media to receive social validation [45].

One uniqueness of the present study is that it measured different types of online activities. This certainly serve as a positive response to the researchers’ call for the need to capture various online activities and its impacts on adolescent well-being [30]. Intervention programs targeting the proper use of SNS among adolescents can be noted in the present study. Educational effort should be implemented for promoting internet literacy, such as negative and positive communication on SNS platforms.

The present study extends the literature by showing the moderating role of gender on the relation between SNS use and self-esteem. Nowadays, adolescent consider SNS as a salient source to obtain social attention and support [91]. They tend to gravitate towards SNS in order to constantly stay connected with their “*friends*”. It is noteworthy that different SNS use explain a moderate amount of variance (i.e., 13%) in self-esteem. This is consistent with past work by Hawi and Samaha [92] who compared gender differences in the relationships between SNSs use and self-esteem (below 10%) among university students in Lebanon. To capture a deeper understanding of mechanism between SNS use and well-being, Saiphoo et al. [24] argued that “*Frequency of SNS use may not be a nuanced enough measure*” (p. 9). Future investigation may explore how these relations differ by the nature of SNS use. Past research found that the impact of SNS use depends on whether individuals are actively or passively engaging with social media content [93,94]. Additionally, more research is warranted to explore how other factors, such as fear of missing out [95] and effortful control [13], are associated with SNS use and self-esteem.

The present study has some limitations. First, a cross-sectional design has been adopted. Therefore, causal inferences about the linkage between online activities and well-being cannot be inferred. Future research may employ experimental or longitudinal design to demonstrate causal evidence. Second, the sample size of the present study was relatively modest (*N* = 193). Yet, the findings were robust as the fit statistics show satisfactory results. Third, findings were based on self-report measures, which might be influenced by social desirability, although confidentiality and anonymity have been highlighted during the data collection process. Lastly, the data were collected based on adolescents. Findings may not be generalized to other populations (e.g., adults, elderly).

## 5. Conclusions

The present study demonstrates how gender moderates the relation between online activities and well-being. Given the prevalence of the SNSs in our daily life, more research in this area is warranted to help us understand how SNSs relate to health and psychological well-being.

## Figures and Tables

**Table 1:** Demographic background of the participants.

	Whole	Male (*n* = 111, 57.5%)	Female (*n* = 81, 42.5%)	
Age	*M* = 13.33, *SD* = 1.58	*M* = 13.53, *SD* = 1.67	*M* = 13.05, *SD* = 1.43	*t* = 2.15, *df* = 185.02, *p* < 0.05
Place of birth				*ꭓ*^2^(1) = 1.03, *df* =1, *p* >.05
Hong Kong	159 (83.2%)	94 (84.7%)	64 (79%)	
Others	33 (16.8%)	17 (15.3%)	17 (21%)	
Receiving financial aids ^a^				*ꭓ*^2^(1) = 0.06, *df* = 1, *p* > 0.05
Yes	116 (60.7%)	69 (62.2%)	49 (60.5%)	
No	75 (39.3%)	42 (37.8%)	32 (39.5%)	
Family structure				*ꭓ*^2^(1) = 0.01, *df* = 1, *p* > 0.05
Intact	131 (69.7%)	76 (68.5%)	55 (67.9%)	
Non-intact	62 (30.3%)	35 (31.5%)	26 (32.1%)	

*Note*. ^a^ Received financial aid from school or government.

**Table 2:** Descriptive statistics and correlations of all variables.

Variable	*M*	*SD*	Skewness	Kurtosis	α	1	2	3	4
1. Information searching	2.43	1.35	0.118	−0.844	0.67	-			
2. Social interaction	3.64	1.51	−0.780	−0.662	0.75	0.54 **	-		
3. Entertainment	2.45	1.06	0.034	−0.156	0.70	0.54 **	0.42 **	-	
4. Self-esteem	2.71	0.45	0.198	0.114	0.78	0.04	0.07	−0.01	-

** *p* < 0.01.

**Table 3:** Gender differences among all variables.

Variable	Male		Female		*t*	*df*	Cohen’s *d*
	*M*	*SD*	*M*	*SD*			
Information searching	2.45	1.35	2.41	1.37	0.19	169	0.03
Social interaction	3.47	1.48	3.85	1.54	−1.64	169	−0.25
Entertainment	2.60	0.98	2.25	1.16	2.17 *	153	0.33
Self-esteem	2.73	0.46	2.67	0.42	0.94	174	0.14

* *p* < 0.05.

**Table 4:** Standardized coefficients of path analysis.

	Estimate	*SE*
Gender → self-esteem	−0.52 **	0.17
Information searching → self-esteem	−0.06	0.11
Social interaction → self-esteem	0.04	0.11
Entertainment → self-esteem	0.04	0.12
Information searching X Gender → self-esteem	−0.05	0.19
Social interaction X Gender → self-esteem	0.53 **	0.20
Entertainment X Gender → self-esteem	−0.11	0.22
*R* ^2^	0.13 **	

** *p* < 0.01.

## Data Availability

Data are available from the author upon request.
